# The association between transient childhood psychotic experiences and psychosocial outcomes in young adulthood: Examining the role of mental disorders and adult attachment

**DOI:** 10.1111/eip.13382

**Published:** 2023-01-16

**Authors:** Lorna Staines, Colm Healy, Ian Kelleher, David Cotter, Annette Burns, Mary Cannon

**Affiliations:** ^1^ Royal College of Surgeons in Ireland, Psychiatry, Education and Research Centre Beaumont Hospital Dublin Ireland; ^2^ Division of Psychiatry Centre for Clinical Brain Sciences University of Edinburgh Edinburgh UK; ^3^ Department of Psychiatry Beaumont Hospital Dublin Ireland; ^4^ Bamford Centre for Mental Health and Well Being Ulster University Coleraine UK

**Keywords:** adult, attachment, attachment, childhood and adolescent, mental disorders, psychosocial outcomes, psychotic experiences

## Abstract

**Aim:**

Evidence suggest individuals with mental disorders and psychotic experiences (PE), even transient PE, show poorer psychosocial outcomes relative to those with mental disorders. The concept of “attachment” is hypothesized as the mechanism by which people seek support in times of need. This can be measured as discrete styles or as positive (low avoidance/anxiety)/negative (high avoidance/anxiety) dimensions. Adult attachment has previously been examined on PE risk factors, but not outcomes. This study aimed to examine the relationship between transient childhood PE and adult psychosocial outcomes, comparing those with and without mental disorders. Second, to examine the role of adult attachment.

**Method:**

Participants (*n* = 103) attended baseline (age 11–13) and 10‐year follow‐up. PE and mental disorders were measured using the Schedule for Affective Disorders and Schizophrenia for School‐aged Children. Attachment and outcomes were measured using self‐report measures. Analysis compared those with PE (with/without mental disorders), and mental disorders without PE, to controls, using linear and Poisson regression.

**Results:**

PE was associated with lower self‐esteem (*β* = −2.28, *p* = .03), perceived social support from friends (*β* = −2.80, *p* = .01), and higher stress in platonic relationships (IRR = 1.64). PE and mental disorders were associated with lower self‐esteem (*β* = −5.74, *p* = .002), higher stress in romantic (IRR = 1.40) and platonic (IRR = 1.59) relationships, general stress (*β* = 5.60, *p* = .006), and mental distress (*β* = 5.67, *p* = .001). Mental disorders alone was not associated with any measure. Adult attachment dimensions attenuated some results.

**Conclusions:**

This paper illustrates the association between transient PE and adult psychosocial outcomes, with & without co‐occurring mental disorders, and demonstrates the role of adult attachment.

## 1 INTRODUCTION

Psychotic experiences (PE) are relatively common, with a prevalence rate of approximately 5%–7% of adults (Linscott & van Os, 2013; van Os et al., 2009), and ~17% of children (Kelleher, Connor, et al., 2012). Evidence shows that PE, even when transient, are associated with long‐term adverse outcomes (Carey et al., 2020; Healy et al., 2018). Mental disorders and PE are often co‐occurring (Healy et al., 2019). Those who report both PE and mental disorders have worse outcomes than those with only mental disorders, including elevated risk of additional subsequent mental disorders (Rimvall, van Os, Rask, et al., 2020), suicide behaviours (Bromet et al., 2017), and increased use of mental health services (Bhavsar et al., 2021). To date less research has focused on non‐clinical outcomes, such as psychosocial outcomes. Trotta et al. (2020) found PE in childhood were associated with poor psychosocial outcomes (loneliness, lower quality of life and higher rates of criminal behaviour) in adulthood, but the wider scope of psychosocial outcomes following PE, are currently under‐examined.

Attachment is hypothesized as an innate psychobiological dependency infants have for care givers, which depending on positive/adverse support from caregivers, develops into positive/negative attachment (Bowlby, 1982). Attachment was originally hypothesized to form in early childhood and remain stable (Ravitz et al., 2010), but more recent evidence has failed to support this (Badovinac et al., 2021; Fearon & Roisman, 2017; Mullen, 2019). Evidence does support that attachment can reliably predict different outcomes; Adult attachment has been linked to differences in self‐esteem, mental health difficulties, and response to treatment (Foster et al., 2007; Kuipers & Bekker, 2012; Mullen, 2019). Attachment can be measured as discrete styles, although more recent evidence supports a dimensional approach (Fearon & Roisman, 2017). Child & adult attachment has previously been shown to mediate the relationship between trauma and rates of PE (Sheinbaum et al., 2014, 2020). Its role in outcomes following PE, are currently unknown.

This paper aims to examine the effect of transient PE on adult psychosocial outcomes, examining the differences between those who report both PE and mental disorders, PE, and mental disorders, compared to controls. Secondly we examined the role of adult attachment.

## 2 MATERIAL AND METHODS

### 2.1 Recruitment and participants

Participants were recruited as part of the Adolescent Brain Development study, [for full details see (Kelleher, Murtagh, et al., 2012)]. Briefly, at baseline 211 participants aged 11–13 (mean age 11.7), were recruited from primary schools and invited for in‐depth clinical interviews. All participants were invited to return for a follow‐up 10 years later. At follow‐up, 103 participants returned [for full details see (Carey et al., 2020)]. Only individuals who completed the adult follow‐up assessment are included within this study.

## 3 MEASUREMENTS

### 3.1 Demographic and clinical information

At baseline demographic information on age and sex were collected. Victimization was based on presence of the adverse experiences of physical abuse, sexual abuse or bullying, (Coughlan et al., 2021). Victimization was a dichotomous variable, using binary (present/not) for physical and sexual abuse, bullying was treated as binary using the threshold of above the median score for distress (4/10) for incidents of bullying.

### 3.2 Mental health assessment

#### 3.2.1 Psychotic experiences

At baseline, participants completed clinical screenings using the Schedule for Affective Disorders and Schizophrenia for School‐aged Children, Present and Lifetime Versions (K‐SADS) (Kaufman et al., 1997). At 10‐year follow‐up participants were interviewed using the psychosis section of the Structured Clinical Interview for DSM‐5 (First et al., 2015) & additional to questions from the SOCRATES instrument (Kelleher & Cannon, 2016).

#### 3.2.2 Mental disorders

At baseline the K‐SADS measure (Kaufman et al., 1997) was used to assess Axis 1 mental disorders, measuring current and lifetime morbidity, classified by the DSM‐IV of Axis I disorders (American Psychiatric Association, 1994). Only individuals who reported experiences of anxiety and post‐traumatic stress disorders, mood disorders, eating disorders, substance disorders, conduct disorder or psychotic disorders were considered to have mental disorders in analysis. Simple phobias were not considered.

### 3.3 Psychosocial outcomes assessment

All psychosocial measures are self‐report measures and were collected at the follow‐up interview.

#### 3.3.1 Self‐esteem

The Rosenberg Self‐Esteem Scale (RSE), is a 10 item measure of global self‐esteem (Robins et al., 2001). The RSE shows good test–retest reliability (*α* = 0.72–0.82) (Gray‐Little et al., 1997).

#### 3.3.2 Social support

The Multidimensional Scale of Perceived Social Support (MPSS) is a 12 item measure of perceived social support good internal reliability (*α* = 0.84–0.92 (Zimet et al., 1990)).

#### 3.3.3 Mental distress

The General Health Questionnaire (GHQ) is a measure of psychiatric impairment and strain (Banks et al., 1980; El‐Metwally et al., 2018). Evidence suggests the GHQ‐12 best measures the unidimensional measure of mental distress (Anjara et al., 2020; Romppel et al., 2013). The measures shows good internal reliability (*α* = 0.81–0.83) (Politi et al., 1994; Winefield et al., 1989).

##### Stress

(1) The Perceived Stress Scale (PSS), is a 10 item measure of the degree to which daily life was stressful in the last month (Cohen et al., 1983). PSS shows good test–retest reliability (*α* = 0.74–0.91) (Lee, 2012).

(2) An adaption of the Stressful life events scale (SLES, version 3.01) (Williamson et al., 2003) was used to measure stress about romantic and platonic (family and friend) relationships. The SLES is a 79‐item binary (Yes/No) questionnaire about a range of stressful life experiences and difficulties. Nine questions relating to romantic relationships and 20 questions relating to platonic relationships. Scores were summed to calculate two measures of stress in relationships; romantic (0–9) and platonic (0–20).

### 3.4 Adult attachment assessment

The revised adult attachment scale (RAAS) is an 18‐item self‐report questionnaire, used to measure adult attachment (Collins & Read, 1990). Attachment dimensions were used within this study. The RAAS shows fair test–retest reliability (*α* = 0.58) (Ravitz et al., 2010).

### 3.5 Statistical analysis

All analysis was conducted using RStudio (R Core Team, 2020). To assess the effects of transient childhood PE in the analysis, recurring PE (*n* = 2) were excluded from all analysis. Included participants were divided into four groups for all subsequent analysis; those with childhood PEs and no mental disorders, childhood mental disorders and no PE, childhood PE and mental disorders, and those who reported neither (controls). Those with PE & mental disorders, mental disorders, & PE were each independently compared to controls. Two sets of psychosocial measures were examined; individual measures relating a participants appraisal of themselves or their lives, and social measures relating to a participants perception of their relationship to others.

Differences in demographic characteristics & attrition analysis were conducted using t‐tests for continuous variables, chi‐squared tests for parametric tests for categorical variables and Kruskal–Wallis H‐test for non‐parametric categorical variables.

Two models were used;

1. Model 1 accounted for the confounding variables of sex and childhood victimization.
2. Model 2 included the confounders of Model 1 and additionally adult attachment anxiety and avoidance.

Linear regression was used for the continuous measures GHQ‐12, MPSS, PSS and the RES. Effect size was reported using the localized measure of Cohen's *f* ^2^ measure, *f* ^2^ ≥ 0.02, *f* ^2^ ≥ 0.15, and *f* ^2^ ≥ 0.35 represent small, medium, and large effect sizes, respectively (Selya et al., 2012). The adapted SLES was used as a count measure and data showed a positive skew. Therefore a Poisson regression was used. Robust estimate and standard errors were calculated (Cameron & Trivedi, 2009). Within this analysis effect size was measured using incident rate ratio (IRR).

## 4 RESULTS

### 4.1 Attrition analysis

No differences between those who returned at follow‐up and those who did not were found in sex (*χ* ^2^ = 0.01, *p* = 1.0), age (*t* = −1, *p* = .2), childhood PE (*χ* ^2^ = 2, *p* = .1), childhood mental disorders (*t* = −.2, *p* = .8), or childhood victimization (*χ* ^2^ = 2, *p* = .2).

### 4.2 Demographic and psychosocial characteristics

In the sample of 103 participants who returned to follow up, two reported recurring PE and were excluded. 29 reported transient PE in childhood, and 58 reported no mental disorders or PEin childhood. Demographic and psychosocial differences were calculated (Table 1).

**TABLE 1:** Demographic and psychosocial differences between individuals reporting PEs and controls

	Childhood PE (*n* = 29)	Controls (*n* = 58)	*χ* ^2^/*t*/*F*	*p* value
Sex, *f* (%)	10 (34.5)	35 (60.3)	*χ* ^2^ = 4	**.04**
Age at follow‐up, mean (SD)	21 (1.1)	21 (1.4)	*t* = 0.9	.4
Victimization, *n* (%)	30 (51.7)	28 (48.3)	*χ* ^2^ = 6	**.01**
RES, mean (SD)	17 (5.8)	21 (6)	*t* = 3	**.003**
PSS, mean (SD)	17 (5.4)	15 (6.8)	*t* = −2	.06
GHQ‐12, mean (SD)	12 (6.3)	11 (5.4)	*t* = −1	.3
MPSS SO, mean (SD)	23 (5.2)	24 (5.3)	*t* = 0.3	.8
MPSS family, mean (SD)	23 (2.9)	23 (4.6)	*t* = 0.1	.9
MPSS friends, mean (SD)	22 (5.5)	25 (3.5)	*t* = 2	**.02**
SLES romance, median (range)	3 (0–7)	2 (0–6)	H = 3	.09
SLES non‐romantic, median (range)	4 (0–10)	3 (0–8)	H = 6	**.02**

Abbreviations: *χ* ^2^, chi‐squared test; *F*, Kruskal–Wallis rank sum test; GHQ‐12, General Health Questionnaire, 12 item; MD, mental disorder; MPSS, multidimensional scale of perceived social support; PE, psychotic experiences; PSS, perceived stress scale; RES, Rosenberg self‐esteem scale; SLES, stressful life events scale; SO, significant other; *t*, *t* test.

Bold is when *p* values are ≤ 0.05.

Compared to controls, those reporting childhood PE were more likely to be male, and report higher rates of victimization (Table 1). In psychosocial outcomes, PE showed higher levels of stress in daily life and platonic relationships, and lower perceived support from friends (Table 1). No significant differences were found in mental distress, perceived social support from family or significant others, or stress in romantic relationships, between PE and control (Table 1).

This sample was further divided based on those with childhood PE (*n* = 14; 13.9%), mental disorders (*n* = 14; 13.9%), both PE and mental disorders (*n* = 15; 14.9%) and controls (*n* = 58; 57.4%), and analysis of demographic and psychosocial differences were conducted (Table 2). In demographic measures, there was a significant difference at a group level in victimization but not age or sex (Table 2). Additionally, there were significant group level differences in perceived social stress (Table 2).

**TABLE 2:** Demographic differences and psychosocial score between individuals reporting PEs and MD, PEs no MD, MD no PEs and controls

	Childhood PEand MD	Childhood PE, no MD	Childhood MD, no PE	Controls	*χ* ^2^/H/F	*p* value
Sex, *f* (%)	4 (26.7)	6 (37.5)	8 (57.1)	35 (60.3)	*χ* ^2^ = 7	.07
Age at follow‐up, mean (SD)	21 (0.96)	20 (1.2)	21 (1.7)	21 (1.4)	*F* = 0.13	.72
Victimization, *n* (%)	12 (80.0)	13 (81.3)	7 (50.0)	28 (48.3)	*χ* ^2^ = 9	**.03**
RES, mean (SD)	18 (6.4)	16 (5)	19 (7.6)	21 (6)	*F* = 3.86	.052
PSS, mean (SD)	16 (6.2)	20 (4.7)	18 (7.3)	15 (6.8)	*F* = 6.86	**.01**
GHQ‐12, mean (SD)	10 (6.4)	14 (6.1)	12 (5.5)	11 (5.4)	*F* = 2.52	.12
MPSS SO, mean (SD)	23 (4.5)	23 (5.7)	24 (6.6)	24 (5.3)	*F* = 0.02	.90
MPSS family, mean (SD)	23 (2.8)	24 (3.0)	21 (7.5)	23 (4.6)	*F* = 1.4	.24
MPSS friends, mean (SD)	21 (6.2)	23 (5.1)	24 (3.6)	25 (3.5)	*F* = 1.73	.19
SLES romance, median (range)	4 (1–7)	2.5 (0–5)	2 (1–7)	2 (0–6)	*H* = 7	.08
SLES non‐romantic, median (range)	5 (0–10)	3 (0–10)	3 (0–11)	3 (0–8)	*H* = 7	.07

Abbreviations: *χ* ^2^, chi‐squared test; *f*, Kruskal–Wallis Rank Sum test; GHQ‐12, General Health Questionnaire, 12 item; *H*, ANOVA; MD, mental disorder; MPSS, multidimensional scale of perceived social support; PE, psychotic experiences; PSS, perceived stress scale; RES, Rosenberg self‐esteem scale; SLES, stressful life events scale; SO, significant other.

Bold is when *p* values are ≤ 0.05.

### 4.3 Longitudinal relationship of childhood PEs and mental disorders on individual outcomes

**FIGURE 1:**
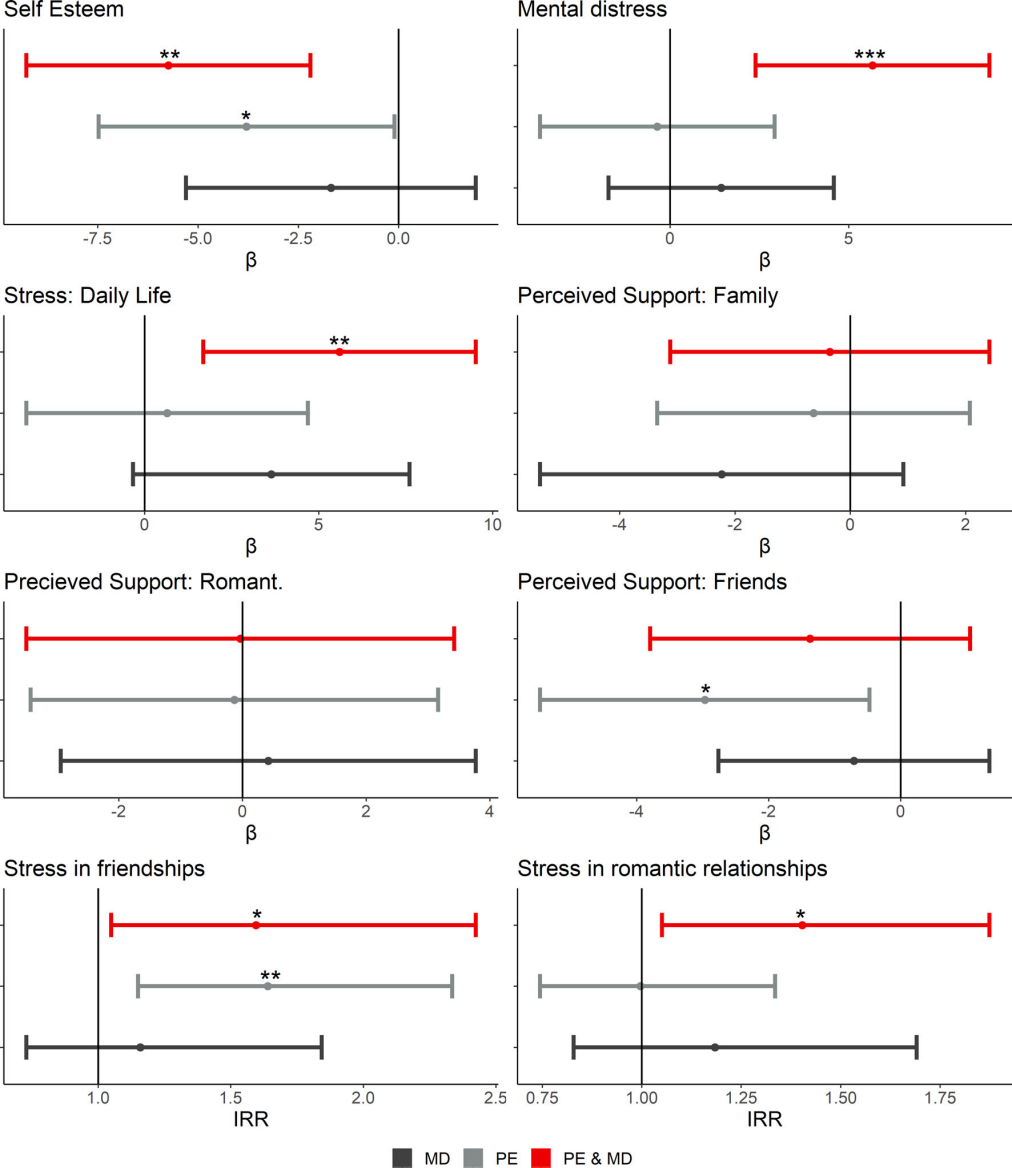
Model 1 psychosocial outcomes of PE, mental disorders and PEs and MD in adulthood. PE, psychotic experiences; MD, mental disorders; PE & MD, psychotic experience and mental disorders group; estimate, β coefficient; IRR, incident rate ratio; Romant., romantic; Romantic/Platonic relation., romantic/platonic relationships

In Model 1 participants reporting PE, with and without mental disorders, were significantly related to lower self‐esteem compared to controls, a medium and small effect size, respectively. (Table 3; Figure 1). Only those reporting both PE and mental disorders in childhood were related with higher levels of general stress and mental distress compared to controls, of small and medium effect size (Table 3; Figure 1). Those reporting mental disorders but not PE in childhood showed no association with later individual psychosocial outcomes, but showed a moderate trend of higher stress levels compared to controls (Table 3; Figure 1). When adult attachment anxiety and avoidance were added (Model 2), the model completely attenuated the association between childhood PE and poor adult self‐esteem, in both the PE and PE and mental disorders group (Table 3). In those reporting PE and mental disorders, worse general stress relative to controls was similarly attenuated. Mental distress remained significantly higher compared to controls even when adult attachment was accounted for, with a small effect size (Table 3).

**TABLE 3:** The role of childhood PEs in adult individual psychosocial outcomes

	Model 1		Model 2	
	*β* (95% CI)	*F* ^2^	*β* (95% CI)	*F* ^2^
PE + MD				
RES	−5.74 (−9.28, −2.20)**	**0.16**	−2.93 (−6.11, 0.25)	0.05
PSS	5.60 (1.69, 9.52)**	**0.12**	2.21 (−1.22, 5.64)	0.03
GHQ‐12	5.67 (2.39, 8.95)***	**0.18**	4.01 (0.70, 7.33)*	**0.09**
PE only				
RES	−3.79 (−7.48, −0.10)*	0.06	−2.34 (−5.46, 0.78)	0.04
PSS	0.65 (−3.40, 4.69)	0.00	−0.93 (−4.05, 2.18)	0.01
GHQ‐12	−0.36 (−3.65, 2.93)	0.00	−1.33 (−4.37, 1.70)	0.01
MD only				
RES	−1.69 (−5.30, 1.93)	0.01	−0.86 (−3.59, 2.02)	0.00
PSS	3.64 (−0.34, 7.61)	0.05	2.36 (−0.55, 5.28)	0.00
GHQ‐12	1.43 (−1.73, 4.59)	0.01	0.811 (−2.00, 3.63)	0.00

*Note*: *p = .05 ; **p = .01; ***p < .001.

Abbreviations: β, estimate; *F* ^2^, Cohen's *F* ^2^ effect size; GHQ‐12, General Health Questionnaire, 12 item; MD, mental disorder; PE, psychotic experiences; PSS, perceived stress scale; RES, Rosenberg self‐esteem scale.

Bold is when *p* values are ≤ 0.05.

### 4.4 Longitudinal relationship of childhood PE and mental disorders on social outcomes

In Model 1, accounting for sex and victimization, childhood PE and mental disorders was associated with elevated levels of romantic and platonic stress, but no difference was found compared to controls in perceived social support (Table 4; Figure 1). Model 2 including adult attachment dimensions accounted for the difference compared to controls for platonic stress, but not the higher rates of stress in romantic relationships reported by this group, with a moderate incidence rate (Table 4). In those who reported PE in childhood but not mental disorders, Model 1 showed PE were associated with lower perceived social support from friends, and stress in platonic relationships, when compared to controls. No other social outcome was associated with only PE (Table 4; Figure 1). Model 2 including adult attachment anxiety and avoidance accounted for the perceived social support in friendship, but it continues to show a general trend. The measure of stress in platonic relationship remained stable with the inclusion of adult attachment, and with a moderate incidence rate ratio (Table 4). The comparison measure of childhood mental disorders with no PE showed no effect on any measure, in either Models 1 or 2. However a trend of higher levels of stress in romantic and platonic relationship was observable, though non‐significant, and showed a moderate incident rate ratio in both Models 1 and 2 (Table 4; Figure 1).

**TABLE 4:** The role of childhood PEs in later social psychosocial outcomes

	Model 1		Model 2	
	*β* (95% CI)	*F* ^2^/IRR	*β* (95% CI)	*F* ^2^/IRR
PE + MD				
MPSS SO	−0.03 (−3.49, 3.43)	0.00	2.07 (−1.36, 5.50)	0.02
MPSS family	−0.35 (−3.12, 2.42)	0.00	0.92 (−1.92, 3.76)	0.01
MPSS friends	−1.37 (−3.80, 1.05)	0.02	0.03 (−2.19, 2.25)	0.00
SLES romance	0.34 (0.05, 0.63)*	1.40	0.31 (0.07, 0.53)*	1.36
SLES non‐romantic	0.47 (0.05, 0.89)*	1.59	0.34 (−0.07, 0.74)	1.40
PE only				
MPSS SO	−0.13 (−3.42, 3.17)	0.00	0.94 (−2.06, 3.95)	0.01
MPSS family	−0.64 (−3.35, 2.07)	0.00	−0.00 (−2.63, 2.62)	0.00
MPSS friends	−2.97 (−5.46, −0.47)*	0.08	−1.94 (−3.96, 0.08)	0.06
SLES romance	−0.00(−0.30, 0.29)	1.00	−0.00 (−0.30, 0.30)	1.00
SLES non‐romantic	0.49 (0.14, 0.85)**	1.64	0.48 (0.12, 0.86)**	1.62
MD only				
MPSS SO	0.42 (−2.94, 3.78)	0.00	1.12 (−1.93, 4.16)	0.01
MPSS family	−2.23 (−5.38, 0.92)	0.03	−1.67 (−4.55, 1.20)	0.02
MPSS friends	−0.71 (−2.76, 1.35)	0.01	−0.26 (−1.95, 1.44)	0.00
SLES romance	0.17 (−0.19, 0.53)	1.18	0.13 (−0.25, 0.51)	1.14
SLES non‐romantic	0.15 (−0.32, 0.61)	1.16	0.15 (−0.31, 0.60)	1.16

*Note*: *p = .05; **p = .01; ***p = .001. MPSS was run as linear regression, SLES was run using Poisson analysis.

Abbreviations: MD, mental disorder; MPSS, multidimensional scale of perceived social support; PE, psychotic experiences; SLES, stressful life events scale; SO, significant other.

Bold is when *p* values are ≤ 0.05.

## 5 DISCUSSION

This paper set out to examine the role of transient childhood PE on outcomes in later life, accounting for the role of adult attachment. Two key findings emerged; (1) Transient PEin childhood, with and without mental disorders, were associated with poorer outcomes in adulthood in the areas of: self‐esteem, general stress, perceived social support, and stress in romantic and platonic relationships. Mental disorders in childhood was not found to be related directly to any adult psychosocial outcomes. (2) Including adult attachment into these models significantly attenuated the relationship between childhood PE and certain adult psychosocial measures.

The primary finding of this paper was the long term association between childhood PE on adult outcomes. Transient PE, even in the absence of co‐occurring mental disorders, was associated with lower self‐esteem and perceived social support from friends, and higher levels of stress in platonic relationships. This study supports indicating that childhood PE are a marker of poor mental health which can show sustained negative trajectories into adulthood (Bhavsar et al., 2021; Bromet et al., 2017; Carey et al., 2020; Healy et al., 2018, 2019; Trotta et al., 2020), showing this association is relevant in psychosocial outcomes, in addition to clinical outcomes.

Mental disorders without PE, were not found to be significantly related to any psychosocial measure, but showed a moderate trend in all measures of stress. One explanation for this may be that childhood mental disorders in the absence of PE may represent a less severe trajectory. Previous research has reported findings similar to this study (Rimvall, van Os, Verhulst, et al., 2020). Alternatively, psychotic experiences have been proposed as a marker of severe psychopathology (Ajnakina et al., 2019; Guloksuz & van Os, 2018; Stochl et al., 2015). While these participants met criteria for a mental disorder, they perhaps had less severe symptoms, which allowed them to resolve prior to adulthood without this adverse effect.

Psychotic experiences and mental disorders in childhood showed particularly poor outcomes compared to controls. PE and mental disorders in childhood was associated with lower self‐esteem and higher rates of general stress, and stress in romantic and platonic relationships in adulthood. This study is in line with previous work, which has shown those with both PE and mental disorders report worse functioning, and higher rates of suicidal ideation, suicidal behaviour, and use of mental health services (Bhavsar et al., 2021; Bromet et al., 2017; Kelleher et al., 2015).

Additionally, PE and mental disorders showed differences in psychosocial outcomes in adulthood, than reported by the PE or mental disorder groups, for example, mental distress and stress in romantic relationships were sustained effects. Similarly the PE group reported lower social support in friends, not found in the PE and mental disorder group. It has been suggested (Bhavsar et al., 2021) that research on PE often treats mental disorders as a confounding variable, rather than as an indicator of a different trajectory.

The second key finding was the inclusion of adult attachment dimensions in the models attenuated some findings. Adult attachment dimensions accounted for the differences between PE and controls in self‐esteem, general stress and perceived social support. Previous research had shown that adult & childhood attachment mediates the relationship between certain risk factors for PE, such as traumatic life events (Gawęda et al., 2018; Sheinbaum et al., 2014, 2020). This study furthers this research by indicating that in adulthood, high attachment anxiety and avoidance may better explain certain psychosocial outcomes, which were previously suggested to be associated with PEs, such as self‐esteem (Gawęda et al., 2012; Hafeez & Yung, 2021). Indeed, the large differences found for several outcomes suggest that adult attachment may be an important, often overlooked, factor in PE research.

## 6 LIMITATIONS

Several limitations should be considered. The sample size was modest in the current study, risking a power issue, and limiting scope of analysis. However, these findings were supported by current literature. Similarly, the attrition rate was high, while no differences in baseline measures were found, it should not be assumed that there are no differences at follow‐up in those who opted not to return. Secondly the psychosocial outcomes were only collected at follow‐up, as were attachment measures. This limited analysis to adult attachment only.

## 7 CONCLUSIONS

Childhood PE, even when transient, are a marker of poor mental health which show sustained associations in many areas of adult life, self‐esteem, perceived social support and stress. This was particularly observable in those who report both PE and mental disorder. Difficulties in self‐esteem, mental distress, stress and social support impact on quality of life (Gayer‐Anderson & Morgan, 2012; Yaribeygi et al., 2017) and should be considered a significant adverse outcome. The adult attachment results suggests that an individual's adult attachment avoidance and anxiety may affect psychosocial outcomes.

## CONFLICT OF INTEREST

The authors declare no conflict of interest.

## Data Availability

The data that support the findings of this study are available from the corresponding author upon reasonable request.
